# Normal age-related changes in left ventricular function: Role of afterload and subendocardial dysfunction

**DOI:** 10.1016/j.ijcard.2016.07.252

**Published:** 2016-11-15

**Authors:** Jehill D. Parikh, Kieren G. Hollingsworth, Dorothy Wallace, Andrew M. Blamire, Guy A. MacGowan

**Affiliations:** a Institute of Cellular Medicine, Newcastle University, Newcastle upon Tyne, United Kingdom; b Newcastle Magnetic Resonance Centre, Newcastle University, Newcastle upon Tyne, United Kingdom; c Institute of Genetic Medicine, Newcastle University, Newcastle upon Tyne, United Kingdom; d Centre for In Vivo Imaging, Newcastle University, Newcastle upon Tyne, United Kingdom; e Dept. of Cardiology, Freeman Hospital, Newcastle upon Tyne, United Kingdom

**Keywords:** Ageing, Magnetic resonance imaging, Mechanics

## Abstract

**Background:**

In normal ageing, both vascular and ventricular properties change, and how these affect left ventricular function is not clear.

**Methods:**

96 subjects (ages 20–79) without cardiovascular disease underwent cardiac magnetic resonance (MR) imaging for measurement of global function, diastolic function (E/A ratio), MR tagging for measurement of torsion to shortening ratio (TSR, ratio of epicardial torsion to endocardial circumferential shortening, with increase in TSR suggesting subendocardial dysfunction relative to the subepicardium), and phase contrast MR imaging measurement of central aortic pulse wave velocity (PWV). The Vicorder device was used to measure carotid to femoral PWV.

**Results:**

Univariate correlations established that the 4 principal age-related changes in the left ventricular function were: 1) diastolic function: E/A ratio (r: − 0.61, p < 0.00001); 2) global systolic function: cardiac output (r: − 0.49, p < 0.00001), 3) structure: end-diastolic volume index (r: − 0.39, p < 0.0001), and 4) systolic strains: TSR (r: 0.49, p < 0.0001). Multiple linear regression analysis showed that age was the dominant factor in predicting changes in cardiac output and E/A ratio (both p < 0.01). Increased TSR was significantly related to reduced cardiac output and end-diastolic volume index (p < 0.05 and p < 0.01 respectively). Measures of vascular stiffness were not significantly related to any of these variables, but increased effective arterial elastance (afterload) was significantly related to reduced E/A ratio (p < 0.05).

**Conclusions:**

In this group of normal ageing subjects, afterload but not vascular stiffness is significantly related to diastolic dysfunction. Increased TSR, suggesting relative subendocardial dysfunction, has a significant role in reductions of cardiac output and end-diastolic volume index.

## 1 Introduction

To understand how ageing with cardiovascular risk factors can lead to heart failure in the elderly, it is firstly important to understand how normal ageing affects left ventricular function. Vascular stiffening increases from the 3rd decade or earlier in normal subjects [1]. This is thought to affect the heart by enhanced arterial wave reflections creating a greater afterload on the heart [2]. To date, studies showing relationships of vascular ageing to changes in left ventricular systolic and diastolic function have concentrated on community-based subjects, in whom there are significant proportions of conditions such as hypertension and diabetes mellitus that will increase vascular stiffness [3], [4], [5]. In normal ageing without cardiovascular disease, the relationships between vascular stiffness may not be as pronounced. A major purpose of this study was therefore to comprehensively study the relationship of normal age-related changes in vascular stiffness and afterload with changes in left ventricular function.

Magnetic resonance imaging can accurately assess changes in left ventricular blood pool volumes with age [6]. Vascular function was assessed by several parameters, each of which has distinguishing features. Pulse wave velocity (PWV) was assessed with the Vicorder device which measures pulse wave velocity from carotid to femoral arteries [7], [8], [9]. PWV was also measured with phase contrast MRI (PC MRI), which measures velocity in the descending thoracic aorta (Central MR PWV). Velocities will be lower in the central vessels, so these measures are not identical [10], [11]. The augmentation index (AI) measures the late systolic wave reflection that poses an afterload on the left ventricle. Arterial elastance is a measure of afterload that is based on the pressure-volume framework and represents the ratio of end-systolic pressure to stroke volume [12]. Potentially the measures of vascular stiffness and afterload differ in that afterload represents the whole of the ejection time whereas vascular stiffness represents a distinct time in late systole when wave reflections might affect left ventricular function [13].

**Fig. 1:**
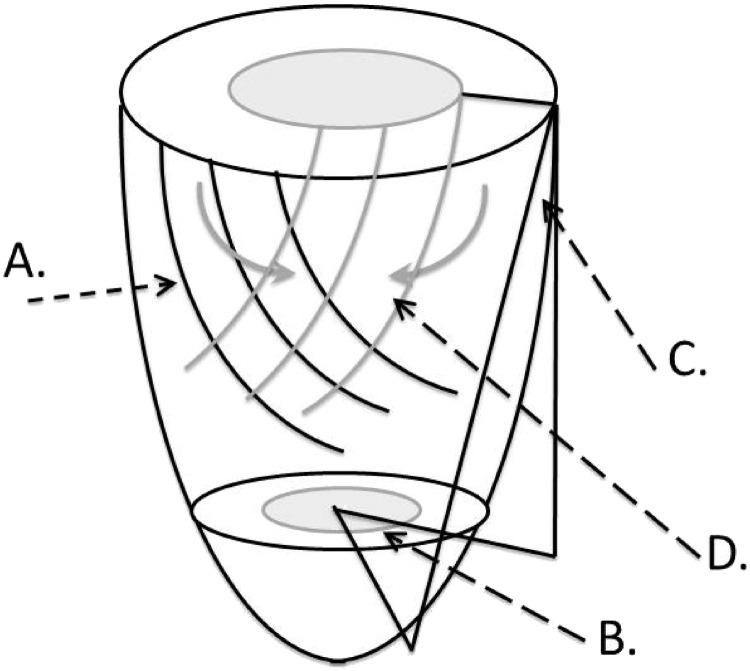
Illustration of epicardial torsion and endocardial circumferential shortening used in the calculation of the torsion to shortening ratio (TSR) and the relationship to subepicardial and subendocardial fibre orientations. Epicardium is black and endocardium grey. A. Obliquely oriented subepicardial fibres produce rotation of the apex with respect to the base (B.) in a counterclockwise direction when looking from the apex to base, which is quantified in terms of the circumferential-longitudinal shear angle (C.). Epicardial torsion acts on the subendocardium with its greater mechanical advantage due to its larger radius, forcing subendocardial fibre bundles to shorten in a direction at almost 90^o^ away from the subendocardial fibre direction (D.). This subepicardial to subendocardial interaction is quantified as the torsion to shortening ratio (TSR), and an increase in the TSR suggests subendocardial dysfunction relative to the subepicardium.

Torsion, and the ratio of torsion to endocardial circumferential shortening (TSR) are also known to increase with normal ageing [14]. TSR is a measure of the subepicardial influence over the subendocardium, with relative dysfunction in the subendocardium increasing TSR (Fig. 1), though its relevance to changes in left ventricular function are not clear. We determined the relationship of measures of vascular stiffness, afterload and torsion to age-related changes in global systolic and diastolic function.

## 2 Methods

### 2.1 Subjects

Ninety-six subjects (41 males, 55 females) aged between 20 and 79 years were recruited into six discrete age bands, with 16 subjects in each decade 20–29, 30–39, 40–49, 50–59, 60–69 and 70–79 years. The subjects were screened with a 12-lead electrocardiogram, fasting lipid profile, and blood pressure measurements. Subjects with hypertension (systolic blood pressure > 150 mm Hg and/or diastolic blood pressure > 90 mm Hg), were excluded from the study, as well as any other cardiovascular diagnosis, diabetes mellitus or dialysis-dependent renal failure, or any treatment with antihypertensive therapy. There were 9 patients that were deemed not suitable for the study after attending for a screening visit. These were 2 patients aged 40–49, 1 50–59, 4 60–69 and 2 70–79. The reasons for failing the screening visit: were 7 had BP > 150 systolic or 90 diastolic, 1 patient was on a thiazide diuretic, and 1 patient had musculoskeletal problems making MR imaging problematic. Informed written consent was obtained for all patients, and this study was approved by a UK National Health Service Research Ethics Committee (NRES Committee North East — Newcastle & North Tyneside 1, reference number 12/NE/0057, and ClinicalTrials.Gov identifier NCT01504828). All subjects had measurements of pulse wave velocity by the Vicorder device and the MRI on the same day within 2 h.

### 2.2 Vicorder based measurements of vascular stiffness

The Vicorder device (Skidmore Medical, UK) is an inflatable cuff-based device that simultaneously measures the upstroke of carotid and femoral pulsations to calculate pulse wave velocity, and has been evaluated extensively by comparing with invasive measurements and other tonometric devices [7], [8], [9]. The Vicorder measurements were performed by trained research nurses. Patients laid on an examination couch, with the head raised to approximately 30°, so that the skin and muscles over the carotid were relaxed. PWV was measured by a cuff placed over the right carotid and the right thigh. The length between the carotid and femoral arteries was done by measuring the length between the suprasternal notch and the mid-point of the thigh cuff. Other measurements with the Vicorder device were done with a cuff placed on the right upper arm. These included oscillatory blood pressure measurement and using a global transfer function central aortic pressures and AI [15].

### 2.3 Central MR PWV

A Philips Achieva 3T scanner and a 6 channel receiver array coil were employed to acquire cardiac MRI data as previously described, and validated against the Vicorder measures of PWV [16]. Briefly, phase contrast (PC) MRI flow data were acquired at two slice locations in descending aorta approximately 10 cm apart, using high temporal resolution sequence (repetition time (TR) = 5 ms; echo time (TE) = 2.9 ms; flip angle (FA) = 10°; number of excitations (NEX) = 1; slice thickness = 8 mm, parallel imaging sensitivity encoding (SENSE) factor 2, field of view (FOV) = 300 mm × 225 mm, reconstructed voxel size = 1.17mm^2^, velocity encoding (V_enc_) = 150 cm/s, 44 phases, breath hold duration ~ 19 s) [17]. The Q-flow analysis package (Philips, ViewForum version 3) was employed for region of interest analysis, to extract time-velocity curves from the PC MRI acquisitions and to estimate precise distance (ΔX) between the two slice locations. The time-velocity curves were then employed to compute transit time (ΔT) using an in-house Matlab based program and determine pulse wave velocity PWV = ΔX/ΔT [17]. Additional scout images were acquired to facilitate positioning of the PC MR acquisitions and to ensure that the slices were positioned perpendicular to aorta at both locations.

### 2.4 Cardiac cine imaging

Details of cardiac cine imaging have been previously reported in detail [18]. Briefly, these include short-axis balanced steady-state free precession images which were acquired covering the left ventricle (FOV = 350 mm, TR/TE = 3.7/1.9 ms, acceleration factor 17, FA 40°, slice thickness 8 mm, 0 mm gap, 14 slices, 25 phases, resolution 1.37 mm). Image analysis was performed using the cardiac analysis package of the ViewForum workstation (Philips) to obtain measures of systolic and diastolic function as previously detailed. The following hemodynamic parameters were derived: effective arterial elastance (Ea = end-systolic pressure (systolic blood pressure × 0.9) / stroke volume normalised to body surface area), end-systolic elastance (Ees = end-systolic pressure / end-systolic volume normalised to body surface area), and ventricular-arterial coupling by the ratio of Ees/Ea. Assessment of diastolic function from cine images was performed by calculating the ratio of peak early and late left ventricular filling rates (E/A ratio), and the early filling percentage was calculated as the volume increase from end-systole to the midpoint divided by the stroke volume and multiplied by 100. The eccentricity ratio (left ventricular mass in g over the end-diastolic volume in ml) was calculated as a measure of concentric remodelling. Longitudinal shortening was determined in the four-chamber view by determining the perpendicular distance from the plane of the mitral valve to the apex in systole and diastole. The myocardial wall thickness at systole and diastole was determined at the same level as the cardiac tagging, and radial thickening was calculated.

### 2.5 Cardiac tagging and regional strains

Tagged short axis images were obtained at the same session. A turbo-field echo sequence with acceleration factor 9 was employed (TR/TE/FA/NEX = 4.9/3.1/10/1, parallel imaging SENSE factor 2, FOV 350 × 350 mm, voxel size 1.37 × 1.37 mm, tag spacing of 7 mm) [18]. The Cardiac Image Modelling package (University of Auckland) was used to analyse the tagging data by aligning a mesh on the tags between the endo- and epicardial contours, and is described in detail elsewhere [14], [18]. The epicardial torsion between the two planes (taken as the circumferential-longitudinal shear angle defined on the epicardial surface) was calculated [19]. Circumferential strain was measured for both the whole myocardial wall and the endocardial third of the wall thickness. The ratio of the peak torsion (in radians) [20], and the peak circumferential strain in the endocardial third of the myocardium (subendocardium, %) was derived and is referred to as the torsion to shortening ratio, TSR (Fig. 1) [18], [20], [21]. The recoil of torsion in early diastole is a measure of active relaxation [22] was expressed as the torsion recoil rate (which is normalised for peak torsion, %/ms).

### 2.6 Data and statistical analysis

The strategy was to determine how measures of vascular function and afterload (MR and Vicorder PWV, AI, and effective arterial elastance) changed with age, and identify which parameters of systolic, diastolic function and strains were most strongly related to age by the Pearson correlation method. We then used the Bonferroni correction procedure for multiple comparisons when comparing measures of vascular stiffness with left ventricular function. Those variables that were significantly related with this univariate analysis were then used in the multiple linear regression analysis to determine independent predictors of the measures of age-related measures of left ventricular function, while additionally accounting for the effects of gender. Comparisons of means between groups were tested using ANOVA. Where appropriate, data were normalised to body surface area. Statistical testing was performed using Matlab, Mathworks, Cambridge, UK, and SPSS version 22. Statistical significance level was taken to be p < 0.05.

## 3 Results

Key subject characteristics are reported in Table 1.

### 3.1 PWV and measures of afterload increase with age

**Fig. 2:**
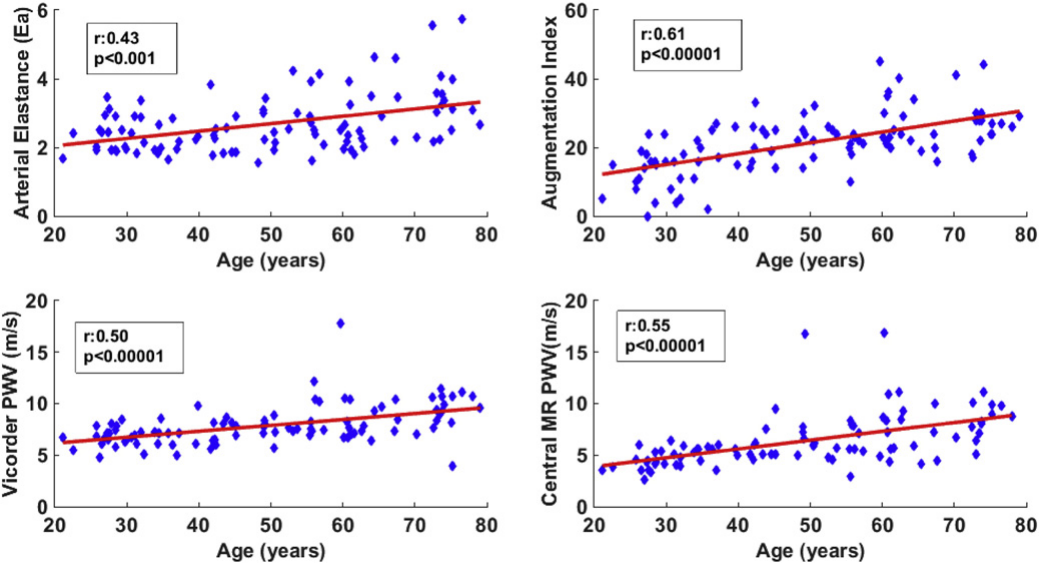
Vascular function and afterload in healthy ageing. Scatter plots describing arterial function with age, along with Pearson's correlation (r) and significance level (p). Arterial elastance, augmentation index, Vicorder PWV and Central MR PWV all increase with age.

PWV increased significantly with age with both techniques (r: 0.55 MRI, and r: 0.50 Vicorder, both p < 0.00001), as shown in Fig. 2 and Table 2. Likewise AI increased with age (Fig. 2 and Table 2, r: 0.61, p < 0.00001). Effective arterial elastance (r: 0.43, p < 0.001, Fig. 2 and Table 2) significantly increased with age, though ventricular–arterial coupling (r: − 0.09, p > 0.05) was unchanged with age.

### 3.2 Left ventricular structure and global systolic function: cardiac output reduces with age

**Fig. 3:**
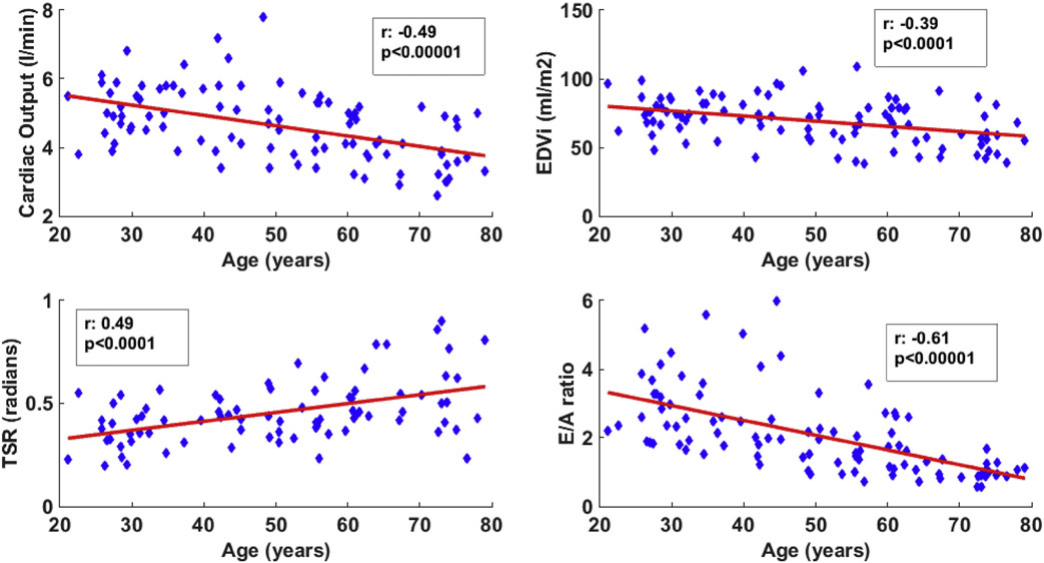
Left ventricular function in healthy ageing. Scatter plots along with Pearson's correlation (r) and significance level (p) demonstrating impaired in left ventricular function in age, with declining cardiac output, end diastolic volume index (EDVi), early to late filling ratio (E/A ratio), and increasing torsion to shortening ratio (TSR).

Cardiac output (r: − 0.49, p < 0.00001, Fig. 3 and Table 2), cardiac index (r: − 0.46, p < 0.0001), end-diastolic volume index (r: − 0.39, p < 0.0001, Fig. 3 and Table 2) and stroke volume index (r: − 0.40, p < 0.0001) all declined with age, while there was no significant change in the ejection fraction (r: 0.11, p > 0.05). Left ventricular mass (r: − 0.20, p < 0.00001) decreased with age, while left ventricular mass /  end-diastolic volume (r: 0.22, p < 0.05) and eccentricity ratio (r: 0.27, p < 0.01) increased respectively. These results demonstrate increased concentric remodelling in ageing hearts.

### 3.3 Systolic torsion and regional strains: increased torsion-related measures with age

Torsion increased with age, with both peak torsion (r: 0.46, p < 0.00001) and TSR (r: 0.49, p < 0.0001, Fig. 3 and Table 2) increasing. Peak endocardial circumferential strain used in the calculation of TSR did not change. Radial thickening increased (r: 0.22, p < 0.05), while longitudinal shortening remained unchanged. Thirteen subjects were excluded from data analysis due to insufficient tag image quality, these include 2 from age group 20–29, 4 from age group 30–39, 3 from age group 40–49, 1 from age group 50–59, 2 from age group 60–69, and 1 from age group 70–79.

### 3.4 Diastolic function is impaired with age

Diastolic function was impaired with increasing age, the peak early filling rate decreased with age (r: − 0.61, p < 0.00001), while late filling rate increased (r: 0.38, p < 0.0001), there was marked decrease in the early filling percentage (r: − 0.57, p < 0.00001) and early to late ventricular filling ratio (E/A ratio) (r: − 0.61, p < 0.00001, Fig. 3 and Table 2) with age. Torsion recoil rate (r: − 0.26, p < 0.05) also declined with age.

### 3.5 Multiple comparisons: increased afterload predicts impaired diastolic function, and increased TSR predicts reduced cardiac output and end-diastolic volume index

The 3 most significant parameters of the left ventricular function related to ageing which were cardiac output, diastolic function (E/A ratio) and systolic strains (TSR), and one measure of the left ventricular structure (end-diastolic volume index) (Fig. 3) were compared to the measures of vascular function and afterload (AI, PWV measured by both Vicorder and MR and arterial elastance, Fig. 2) using the Bonferroni correction for multiple comparisons (Table 2). Effective arterial elastance and AI were significantly related to the cardiac output, end-diastolic volume index and E/A. Central MR PWV was significantly related to only E/A. Vicorder PWV velocity was not significantly related to any of these 4 parameters.

Based on the outcomes of the analysis in Table 2, we then used those significantly related variables to determine independent predictors with multiple linear regression (Table 3, Table 4). We avoided putting closely related variables in the same model, which were cardiac output, end-diastolic volume index and arterial elastance, all of which are intrinsically related to stroke volume. These analyses showed that age alone was the strongest predictor of changes in cardiac output and E/A ratio, though end-diastolic volume index and TSR were not independently related to age. Females had a significantly greater reduction in cardiac output (Table 3A). Increased TSR was significantly related to reduced cardiac output and end-diastolic volume index (Table 3A–C). Lower values of end-diastolic volume index were significantly related to greater impairment of diastolic function (Table 3C). AI was not significantly related to any of the 4 parameters. As Central MR PWV was significantly related to E/A ratio with the univariate analysis, the multiple linear regression analysis for E/A as dependent variable was repeated with Central MR PWV replacing AI, though this was also not significantly independently related to the E/A ratio (Table 4B). E/A ratio was however significantly independently related to effective arterial elastance (Table 4C).

## 4 Discussion

We have demonstrated that in a group of normal subjects without cardiovascular diagnoses of a wide range of ages using state of the art MR imaging that a) with normal ageing there is development of abnormalities of left ventricular function characterised by abnormal diastolic function and reduced cardiac output, b) these parameters of normal ageing are not significantly related to 3 measures of vascular stiffness, though diastolic function is related to afterload and also reduced end-diastolic volume index, c) females have greater reduction in cardiac output with ageing, and d) reductions in cardiac output and end-diastolic volume index are significantly related to increased TSR.

### 4.1 Afterload and the heart

In animal experiments it is well recognized that afterload affects relaxation of left ventricular myocardium, with higher afterload resulting in impaired relaxation [23]. Furthermore, studies in intact dogs have shown that the timing of the afterload significantly influences the magnitude of effects on left ventricular relaxation, with later loading during late ejection having greater effects on relaxation [24]. Whereas these are acute studies and the human counterpart of these studies relate to years of altered loading, they nevertheless seem to be clinically relevant. Arterial wave reflections due to increased vascular stiffness which would increase afterload on the heart in late ejection predict cardiovascular events in a large population of community based subjects [25]. The current study indicates that in normal ageing increased afterload is significantly associated with impaired diastolic function, though there is no significant relationship between 3 measures of vascular stiffening and any of the age-related measures of left ventricular function. Other studies have demonstrated significant relationships between measures of vascular stiffness and left ventricular function. One potential reason for this apparent discrepancy is that previous studies have not specifically studied normal subjects [3], [4], [5], and so have included a significant proportion of patients with hypertension and diabetes in whom vascular stiffness would be expected to be increased, thus increasing the effect on the left ventricular function. Nevertheless, this is probably not the only reason. Canepa et al. [26] have shown that in a large study with both normotensive and hypertensive subjects arterial elastance was significantly related to the E/A ratio (measured with echocardiography) only in normotensives and not in hypertensives. Thus, whereas we have comprehensively examined vascular stiffness with 3 different techniques, we cannot exclude a significant relationship with vascular stiffness and diastolic function in normal ageing in a larger study population. The E/A ratio measured with echocardiography measures the velocity through the mitral valve, whereas MRI measures the filling rates of the left ventricle, which are not necessarily the same measurement. The position of the Doppler measurement of E/A ratio has a significant impact on the E/A ratio, with relatively higher values of the peak E velocity as measurements are moved towards the left ventricle from the left atrium [27]. Consistent with that, a direct comparison of E/A ratios between MRI and echocardiography shows higher values for the E/A ratio with MRI [28]. We do not know if measuring diastolic function with other techniques would have found a significant relationship between vascular stiffness and left ventricular function.

### 4.2 Determinants of reduced cardiac output and end-diastolic volume: increased TSR

Torsion is the result of obliquely orientated subepicardial fibres exerting a mechanical advantage over the inner layers by virtue of their greater radius (Fig. 1). Subendocardial fibres, which are oriented obliquely in the opposite direction, oppose subepicardial fibre direction shortening and so reduce epicardial torsion to some extent [29]. Whereas maximal shortening in the human subepicardium is in the direction of the obliquely oriented subepicardial fibres (fibre direction shortening), in the subendocardium it is very close to the direction of circumferential shortening, as a consequence of the outer layers causing shortening in the inner layers in directions at almost 90^o^ to the fibre direction (cross fibre shortening) [29]. Thus, the ratio of epicardial torsion to endocardial circumferential shortening is a measure of the subepicardial influence over the subendocardium, with relative dysfunction in the subendocardium increasing TSR.

Increases in torsion and TSR are a significant abnormality of left ventricular ageing detected in this study. TSR is significantly related to both reduced cardiac output and end-diastolic volume index, though is not related to measures of vascular stiffening or afterload. This last point appears to agree with previous studies looking at load sensitivity of torsion. Studies in human transplant recipients who had radio opaque markers implanted at the time of transplant to quantify strains radiologically show that torsion is quite insensitive to increased afterload [30], and studies in isolated canine hearts have shown that torsion is less afterload dependent than other strains [31]. Lumens and colleagues [32] have also seen increases in torsion and TSR with normal ageing. They suggested that this increase in TSR may be due to subclinical endocardial ischaemia or fibrosis. It seems unlikely that in our cohort of normal subjects without cardiovascular disease should have subendocardial ischaemia from the 4th and 5th decades onwards, which is when torsion and TSR increase. Furthermore, whereas myocardial fibrosis does develop in older subjects, the rate at which it develops is slow (1% per 10 years in males) so again this seems unlikely in our subjects with less than 50 years of age [33]. However, Campbell et al. [34] have provided an innovative explanation for age-related increases in torsion. They have shown in ageing rats that there is prolongation of the decay time of the calcium transients, and that this is greatest in the epicardium. Furthermore, this was associated with a reduction in troponin I phosphorylation in the aged epicardial myocytes. Thus, changes directly within the left ventricle seem responsible for the increased torsion seen with increasing age, and our data show that this contributes to reduced cardiac output and end-diastolic volume index.

Several studies recently have highlighted the significance of reduced end-diastolic volume in older age. Cheng and colleagues [35] have shown that end-diastolic volume reduces by − 0.8 ml per year, and associated with this is a reduction in stroke volume. Fujimoto et al. [36] have shown reduction in end-diastolic volume in normal subjects over 65 years, similar to our findings. Wohlfahrt et al. [37] have shown that in a longitudinal study over 4 years that increases in resistive and pulsatile afterload were related to reductions in end-diastolic volume over the follow-up period, demonstrating a link between left ventricular volume loss and afterload, which in turn mediates left ventricular stiffening. The current study also adds to these findings by demonstrating a significant relationship of reduced volume with impaired diastolic function. It would be, important to perform the current study on a longitudinal basis to examine further the relationship between age-related left ventricular volume loss, torsion and afterload.

### 4.3 Limitations

This is a cross-sectional study, and longitudinal studies will be more powerful at detecting mechanisms of age-related left ventricular dysfunction. We cannot rule out that in a larger study population that measures of vascular stiffness would be significantly related to left ventricular function, even though we have comprehensively examined the role of vascular stiffness with 3 different methods. Likewise we cannot rule out whether another imaging method of measuring diastolic function would have produced a significant relationship. Those limitations must be balanced against our ability to comprehensively and accurately assess multiple aspects of the left ventricular systolic and diastolic function, left ventricular structure and vascular stiffness, which are not possible with other imaging techniques. None of the subjects in this study had a diagnosis of hypertension, and indeed there is a relatively small age-related increase in systolic blood pressure in this study. Recently and after the completion of this study, it has been suggested that the thresholds to diagnose hypertension should be lowered [38], so that in the future some of this study's subjects would be considered to have a diagnosis of hypertension. Furthermore, we have not evaluated our study subjects for diagnoses such as masked hypertension, or nocturnal hypertension.

### 4.4 Conclusions

Ageing in this cohort of normal subjects without cardiovascular diagnoses is characterised by 2 principal effects on the left ventricular function — impaired diastolic function and reduced cardiac output. Impaired diastolic function is not related to measures of vascular stiffening, but is related to increased afterload and also reduced end-diastolic volume. Increased TSR, suggesting relative subendocardial dysfunction, is significantly related to reduced cardiac output and reductions in end-diastolic volume. These data provide important information about normal ageing, and will help determine how ageing contributes to heart failure in later life.

## Funding and conflicts of interest

This study was supported by the British Heart Foundation Clinical Leave Research Fellowship to GMacG (FS/11/89/29162). No other conflicts of interest.

## Figures and Tables

**Table 1:** Key subject characteristics and parameters.

Age group	20–29	30–39	40–49	50–59	60–69	70–79
Age (years)⁎	26.9 ± 2.3	33.9 ± 2.8	44.8 ± 3.2	54.9 ± 3	63.1 ± 2.6	74.3 ± 2.2
Female/male (N)	11/5	9/7	9/7	10/6	7/9	9/7
Weight (kgs)	76.1 ± 16.5	75.1 ± 12	78.8 ± 9.2	76.3 ± 15.6	74.4 ± 18.7	68.5 ± 13.6
Height (cms)	170.8 ± 8.4	172.2 ± 9.6	174.6 ± 11.8	172.4 ± 9	170.4 ± 10.4	164.4 ± 10.1
BMI (kg/m^2^)	26.1 ± 5.8	25.3 ± 2.9	26 ± 3.4	25.6 ± 4	25.3 ± 4.4	25.2 ± 2.6
Body surface area (m^2^)	1.8 ± 0.2	1.8 ± 0.2	1.8 ± 0.2	1.8 ± 0.2	1.8 ± 0.2	1.7 ± 0.2
Heart rate (beats/min)	59.4 ± 7.7	59.6 ± 8	58.8 ± 7.7	60.2 ± 10.2	56.4 ± 9.7	61.4 ± 11.5
Cholesterol (mmol/l)⁎	4.6 ± 0.9	4.5 ± 0.8	4.7 ± 0.9	4.8 ± 0.7	4.8 ± 0.6	5.3 ± 1.1
Triglycerides (mmol/l)	0.93 ± 0.45	0.95 ± 0.61	0.9 ± 0.34	0.86 ± 0.64	1.02 ± 0.47	1.07 ± 0.38
HDL (mmol/l)	1.5 ± 0.4	1.7 ± 0.3	1.5 ± 0.4	1.8 ± 0.6	1.6 ± 0.5	1.6 ± 0.4
LDL (mmol/l)⁎	2.6 ± 0.8	2.4 ± 0.7	2.8 ± 0.7	2.7 ± 0.7	2.8 ± 0.5	3.2 ± 0.9
Systolic pressure (mm Hg)⁎	120.4 ± 10.5	127.4 ± 10.2	124.0 ± 9.0	124.4 ± 7.4	127.1 ± 14.8	136.7 ± 10.8
Diastolic pressure(mm Hg)	64.9 ± 6.9	71.3 ± 8	68.1 ± 6.8	69.1 ± 9.2	68.3 ± 7.6	71.8 ± 7.8
Pulse pressure (mm Hg)⁎	55.4 ± 5.9	55 ± 5.3	55.9 ± 5	55.3 ± 7.4	58.8 ± 10.3	64.9 ± 7.5
Mean arterial pressure (mm Hg)⁎	87.1 ± 8.2	93.5 ± 7.7	91.9 ± 8.5	93 ± 8.1	93.1 ± 10.4	99.3 ± 10.2
Aortic systolic pressure (mm Hg)⁎	113.7 ± 9.3	120.2 ± 8.5	121.1 ± 8.6	122 ± 7.9	124.7 ± 14.3	134.7 ± 11.1
Aortic pulse pressure (mm Hg)⁎	49.4 ± 4.7	49.9 ± 6.5	53.1 ± 4.7	52.9 ± 7.7	56.4 ± 9.9	62.9 ± 7.9

BMI: body mass index, HDL: high density lipoprotein, LDL: low density lipoprotein.

⁎ ANOVA significance p < 0.05.

**Table 2:** Pearson's correlation coefficient (top half of table) and significance level (lower half of table). Age relationships in the first column are uncorrected p values, and for the multiple comparisons between vascular and ventricular parameters the Bonferroni correction was used (p ≤ 0.0018 for 28 comparisons with significant comparisons highlighted in bold).

	Pearson correlation coefficient between different parameters								
r	Age	CO	EDVi	TSR	E/A	Arterial Elastance	AI	PWV-Vicorder	Central MR PWV
Age									
CO	-0.49								
EDVi	-0.39	**0.63**							
TSR	0.49	**-0.44**	**-0.48**						
E/A	-0.61	0.33	**0.48**	-0.43					
Arterial elastance	0.43	**-0.60**	**-0.87**	0.42	**-0.48**				
AI	0.61	**-0.37**	**-0.26**	0.26	**-0.36**	0.19			
PWV-Vicorder	0.50	-0.22	-0.25	0.15	-0.22	0.35	**0.39**		
Central MR PWV	0.55	-0.36	-0.18	0.26	**-0.42**	0.25	**0.43**	0.23	

	Adjusted significant level: p ≤ 0.0018 (28 comparisons) to account for Bonferroni corrections								
Age									
CO	< 0.00001								
EDVi	< 0.0001	**< 0.00001**							
TSR	< 0.00010	**< 0.00100**	**< 0.00001**						
E/A	< 0.00001	< 0.05000	**< 0.00001**	< 0.01000					
Arterial elastance	< 0.00100	**< 0.00001**	**< 0.00001**	< 0.01000	**< 0.00010**				
AI	< 0.00001	**< 0.00100**	**< 0.00001**	< 0.01000	**< 0.00100**	0.05874			
PWV-Vicorder	< 0.00001	0.47589	< 0.05	1	0.53237	< 0.01000	**< 0.00100**		
Central MR PWV	< 0.00001	< 0.01000	< 0.01	0.36754	**< 0.00100**	0.25182	**< 0.00010**	0.48866	

CO: cardiac output, EDVi: end diastolic volume index, E/A: early to late filling ratio, TSR: torsion to shortening ratio, AI: augmentation index, PWV: pulse wave velocity, MR: magnetic resonance.

**Table 3:** Multiple linear regression analysis with dependent variables cardiac output (A), end-diastolic volume index (B), and TSR (C). Significant values highlighted in bold.

A. Dependent variable: Cardiac output	Beta	t	P =
(Constant)		11.836	0.000
Gender	− 0.201	− 2.116	**0.038**
Age	− 0.388	− 2.682	**0.009**
E/A ratio	− 0.022	−.190	0.850
AI	− 0.044	−.365	0.716
TSR	− 0.242	− 2.334	**0.022**
R^2^ = 0.297			

B. Dependent variable: End diastolic vol. index	Beta	t	P =
(Constant)		9.326	0.000
Gender	− 0.085	− 0.921	0.360
Age	− 0.142	− 1.007	0.317
E/A ratio	0.294	2.574	**0.012**
AI	0.035	0.300	0.765
TSR	− 0.330	− 3.255	**0.002**
R^2^ = 0.329			

C. Dependent variable: TSR	Beta	t	P =
(Constant)		4.111	0.000
Gender	0.077	0.753	0.454
Age	0.234	1.485	0.142
AI	− 0.069	− 0.551	0.583
Cardiac output	− 0.269	− 2.334	**0.022**
E/A ratio	− 0.168	− 1.379	0.172
R^2^ = 0.218			

E/A ratio: ratio of early to late left ventricular filling, AI: augmentation index, TSR torsion to shortening ratio,

**Table 4:** Multiple linear regression analysis with E/A ratio as dependent variable. In (A) AI is included in model, in (B) central MR PWV, and in (C) effective arterial elastance. Significant values highlighted in bold.

A. Dependent variable: E/A ratio	Beta	t	P =
(Constant)		4.863	0.000
Gender	− 0.037	− 0.395	0.694
Age	− 0.572	− 4.351	**0.000**
AI	0.026	0.225	0.823
TSR	− 0.142	− 1.379	0.172
Cardiac output	− 0.021	− 0.190	0.850
R^2^ = 0.340			

B. Dependent variable: E/A ratio	Beta	t	P =
(Constant)		4.187	0.000
Gender	− 0.060	− 0.576	0.566
Age	− 0.499	− 3.544	**0.001**
Cardiac output	− 0.006	− 0.052	0.958
Central PWV	− 0.070	− 0.567	0.573
TSR	− 0.144	− 1.266	0.210
R^2^ = 0.301			

C. Dependent variable: E/A ratio	Beta	t	P =
(Constant)		11.203	0.000
Gender	− 0.030	−.0334	0.740
Age	− 0.458	− 3.538	**0.001**
TSR	− 0.071	− 0.704	0.483
Ea	− 0.234	− 2.281	**0.025**
AI	− 0.020	− 0.172	0.863
R^2^ = 0.380			

AI: augmentation index, TSR: torsion to shortening ratio, PWV: pulse wave velocity, Ea: effective arterial elastance.
